# Val43 residue of NsrR is crucial for the nitric oxide response of *Salmonella* Typhimurium

**DOI:** 10.1128/spectrum.03024-23

**Published:** 2023-12-06

**Authors:** Hee Jeong Park, Hye Won Jeong, Choa Lee, Mi Rae Lee, Hojung Choi, Eungseok Kim, Iel Soo Bang

**Affiliations:** 1 Department of Microbiology and Immunology, Chosun University School of Dentistry, Gwangju, Republic of Korea; 2 Department of Biological Sciences, College of Natural Sciences, Chonnam National University, Gwangju, Republic of Korea; McGill University, Ste-Anne-de-Bellevue, Quebec, Canada

**Keywords:** flavohemoglobin Hmp, nitric oxide, NsrR, *Salmonella *Typhimurium

## Abstract

In pathogenic bacteria, the flavohemoglobin Hmp is crucial in metabolizing the cytotoxic levels of nitric oxide (NO) produced in phagocytic cells, contributing to bacterial virulence. Hmp expression is predominantly regulated by the Rrf2 family transcription repressor NsrR in an NO-dependent manner; however, the underlying molecular mechanism in enterobacteria remains poorly understood. In this study, we identified Val43 of *Salmonella* Typhimurium NsrR (StNsrR) as a critical amino acid residue for regulating Hmp expression. The Val43-to-Ala-substituted mutant NsrR isolated through random and site-directed mutagenesis showed high binding affinity to the target DNA irrespective of NO exposure, resulting in a severe reduction in *hmp* transcription and slow NO metabolism in *Salmonella* under NO-producing conditions. Conversely, the Val43-to-Glu-substituted NsrR caused effects similar to *nsrR* null mutation, which directed *hmp* transcription and NO metabolism in a constitutive way. Comparative analysis of the primary sequences of NsrR and another NO-sensing Rrf2 family regulator, IscR, from diverse bacteria, revealed that Val43 of enterobacterial NsrR corresponds to Ala in *Pseudomonas aeruginosa* or *Streptomyces coelicolor* NsrR and Glu in enterobacterial IscR, all of which are located in the DNA recognition helix α3. The predicted structure of StNsrR in complex with the *hmp* DNA suggests dissimilar spatial stoichiometry in the interactions of Val43 and its substituted residues with the target DNA, consistent with the observed phenotypic changes in StNsrR Val43 mutants. Our findings highlight the discriminative roles of the NsrR recognition helix in regulating species-specific target gene expression, facilitating effective NO detoxification strategies in bacteria across diverse environments.

**IMPORTANCE:**

The precise regulation of flavohemoglobin Hmp expression by NsrR is critical for bacterial fitness, as excessive Hmp expression in the absence of NO can disturb bacterial redox homeostasis. While the molecular structure of *Streptomyces coelicolor* NsrR has been recently identified, the specific molecular structures of NsrR proteins in enterobacteria remain unknown. Our discovery of the crucial role of Val43 in the DNA recognition helix α3 of *Salmonella* NsrR offers valuable insights into the Hmp modulation under NO stress. Furthermore, the observed amino acid polymorphisms in the α3 helices of NsrR proteins across different bacterial species suggest the diverse evolution of NsrR structure and gene regulation in response to varying levels of NO pressure within their ecological niches.

## INTRODUCTION

Bacteria are frequently exposed to antimicrobial radical nitric oxide (NO) and NO-mediated reactive nitrogen species produced in ecological nitrogen cycles. In addition, phagocytic cells of the animal innate immune system generate cytotoxic levels of NO, which can easily diffuse into engulfed bacteria and damage various bacterial macromolecules, leading to abnormal bacterial metabolism and subsequent bacteriostasis (1). Therefore, evading NO-mediated nitrosative stress is a crucial virulence determinant for intracellular pathogens, including *Salmonella enterica*, as it allows them to persist in NO-producing animal hosts (2, 3). To counteract nitrosative stress, many bacteria have evolved a conserved gene *hmp* encoding an ancestral globin, flavohemoglobin Hmp (4). This globin acts to metabolize the NO that enters the bacterial cell, and thus, mutant bacteria lacking the *hmp* gene are highly susceptible to nitrosative stress and exhibit reduced virulence (5, 6). However, the precise mechanisms by which Hmp and its regulation contribute to the bacterial response to NO stress remain to be fully understood.

In *S. enterica* and many other species in the β and γ proteobacteria, the expression of flavohemoglobin Hmp is regulated by the [Fe-S] cluster-containing protein NsrR (5, 7). NsrR functions as a transcriptional repressor, binding to specific DNA sequences around promoters of a set of target genes, including *hmp*. However, upon exposure to NO, NsrR derespresses *hmp* transcription (8). The repression of *hmp* transcription by NsrR under conditions without nitrosative stress is crucial for bacterial fitness, as the overexpression of the flavohemoglobin in the absence of NO can disrupt bacterial redox homeostasis (3, 9, 10). These characteristics highlight the role of NsrR as a master regulator of bacterial gene regulation specifically involved in NO detoxification.

The Rrf2 family protein NsrR contains a [Fe-S] cluster coordinated by three cysteine residues in the C-terminal region (11). Although there have been some conflicting observations regarding the specific forms of the [Fe-S] cluster ([2Fe-2S] or [4Fe-4S]) in purified NsrR proteins from bacterial species such as *Neisseria gonorrhea*, *Bacillus subtilis*, and *Streptomyces coelicolor*, mutational studies on conserved cysteine residues have demonstrated the essential role of the [Fe-S] cluster in regulating target gene transcription (12 – 14). Nitrosylation of the [Fe-S] cluster destabilizes the DNA binding affinity of the NsrR to its target genes, thereby initiating the expression of proteins involved in NO metabolism or alleviating NO stress (13 – 15). Additionally, the first crystal structure of NsrR protein has been identified with NsrR of a Gram(+) *S. coelicolor* (ScNsrR) and revealed a dimeric form of ScNsrR stabilized by a [4Fe-4S] cluster, with the ability of NO to displace a ligand and affect DNA binding ability (16).

Nevertheless, the variation in consensus NsrR-binding DNA sequences across bacterial species and the limited availability of defined molecular structures of NsrR, except for *S. coelicolor*, have left the molecular mechanisms underlying NO sensing through NsrR in enterobacterial species and the specific signal transduction in response to NO largely unexplored. In this study, we aimed to investigate the molecular characteristics of enterobacterial NsrR using mutation analysis on *S. enterica* serovar Typhimurium (*S*. Typhimurium) NsrR. By identifying important amino acid residues involved in regulating *hm*p transcription and subsequent bacterial NO metabolism, this study contributes to understanding NsrR-mediated NO sensing in enterobacteria.

## RESULTS

### Identification of mutant NsrR repressing *hmp* transcription under nitrosative conditions

**Fig 1:**
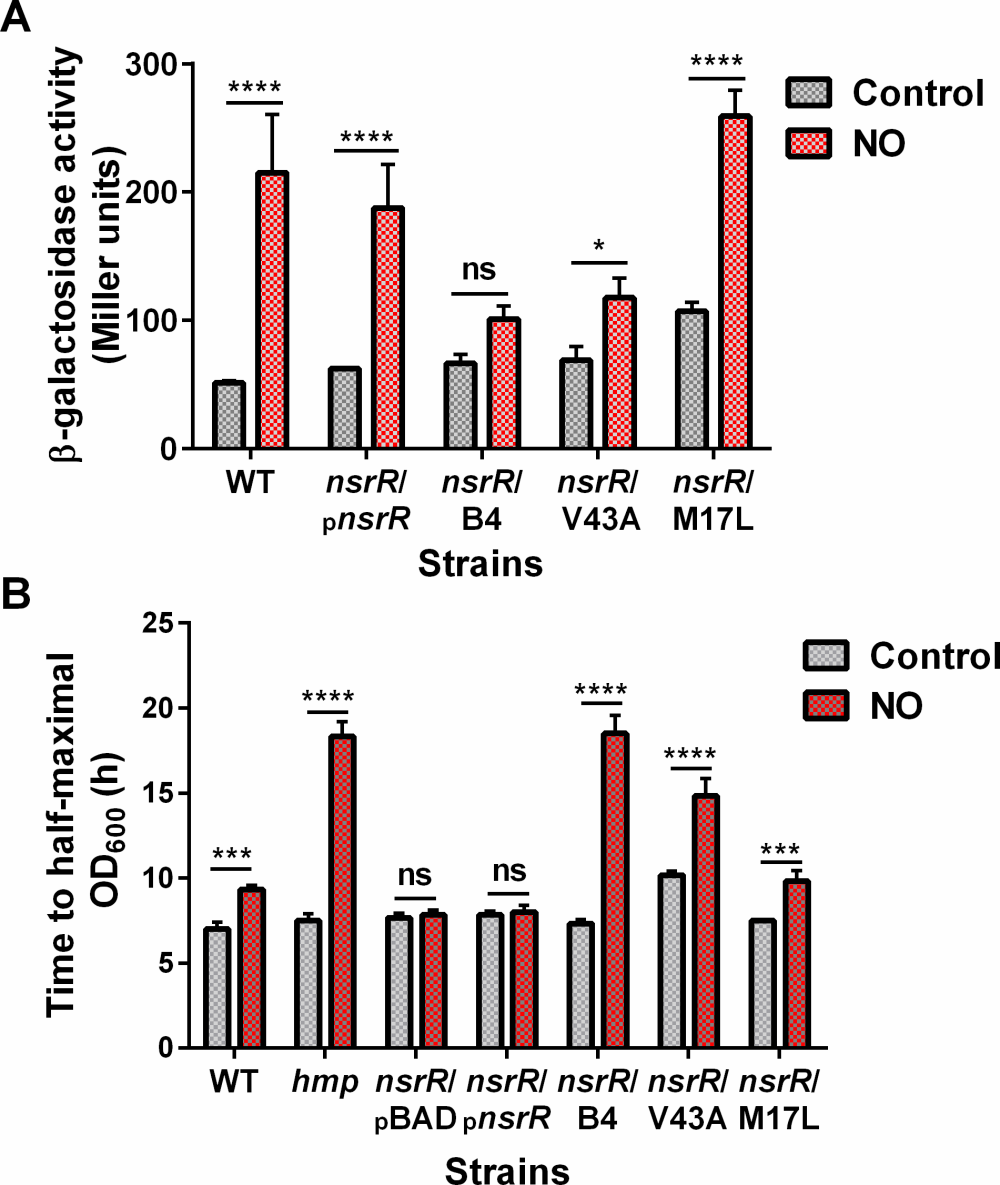
Mutant *nsrR* clones regulating NO-mediated induction of *hmp* transcription. (**A**) β-galactosidase activities of *S*. Typhimurium strains containing the *hmp-lacZ* transcriptional fusion and harboring either mutant or WT *nsrR* on a plasmid. The strains were cultured to log-phase (optical density at 600 nm [OD_600_ ]= 0.5) in E glucose (EG) media (control). Then, half of the culture was supplemented with GSNO (500 μM) and incubated for an additional 1 h (NO) before the β-galactosidase assay. (**B**) Bacterial growth is shown as the mean time to half-maximal OD_600_ ± SD, determined from three independent experiments measuring the growth curve of each strain cultured in EG media with or without GSNO (500 μM) for 24 h. *P  <  0.05, ***P  <  0.001, and ****P  <  0.0001, as determined by two-way analysis of variance; ns, not significant.

To identify amino acid residues of NsrR that play a major part in derepressing *hmp* transcription under NO stress, we conducted a screening of mutant NsrR that could constitutively bind to the *hmp* promoter, leading to the repression of *hmp* transcription regardless of NO exposure. We performed random mutagenesis on the *nsrR* structural gene within a plasmid and transformed these mutants into *S*. Typhimurium carrying the *hmp-lacZ* chromosomal operon fusion. We evaluated the effects of over a thousand transformants on both growth and LacZ activity by replica plating them on solid media containing the NO congener S-nitrosoglutathione (GSNO) (Fig. S1). Among these transformants, 27 exhibited repressive phenotypes, affecting both *hmp* transcription and bacterial growth on the GSNO plates. We further subjected these selected transformants to the measurement of β-galactosidase activity and growth rate in GSNO-containing cultures (Fig. S2 and S3). Interestingly, approximately half of these transformants did not significantly reduce *hmp* transcription, and only one transformant showed GSNO susceptibility. This observation suggests oxygen-level dependence in NsrR-mediated gene regulation, including *hmp* transcription. Figure 1 depicts *S*. Typhimurium expressing the *nsrR* mutant clone B4, which exhibited the most effective repression of *hmp* transcription and bacterial replication under nitrosative conditions. Compared with the wild-type (WT) NsrR, the mutant NsrR B4 caused a significant reduction in β-galactosidase activity of *hmp-lacZ* and bacterial growth rate. These results demonstrate that mutations in NsrR B4 could repress *hmp* transcription in *S*. Typhimurium in response to NO and that this repression is physiologically relevant to the observed bacterial growth retardation under nitrosative stress conditions. DNA sequence analysis revealed that the mutant clone B4 contained two mutations: Met17 to Lys and V43 to Ala (V43A). Site-directed mutagenesis experiments for each single mutation indicated that the V43A mutation primarily accounted for the repression effect observed in the B4 clone on *hmp* transcription.

### V43A mutant NsrR binds to the *hmp* promoter with higher affinity than WT NsrR

**Fig 2:**
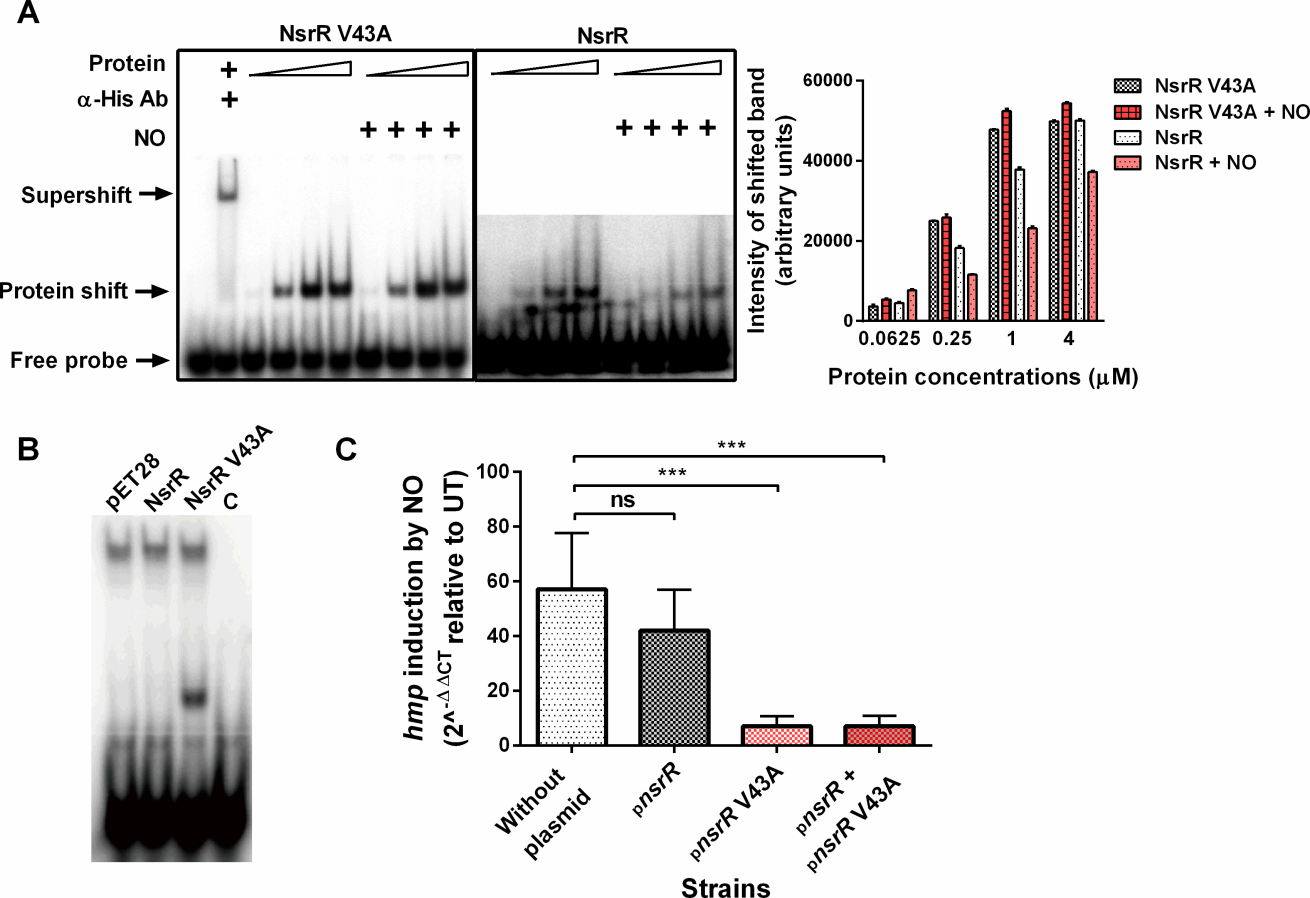
Enhanced binding affinity of V43A mutant NsrR to *hmp* target DNA. (**A and B**) EMSA of WT and V43A mutant NsrR proteins with the *hmp* probe DNA. (**A**) ^32^P-labeled *hmp* probe DNA was incubated with purified His-tagged WT NsrR (right) and V43A mutant NsrR (left) proteins (0.0625, 0.25, 1, and 4 μM for each protein), under the condition described in Materials and Methods, followed by PAGE analysis. Anti-His antibodies were added for supershift assay. Spermine NONOate (1 mM) was used as an NO donor. Intensities of NsrR protein-shifted probes were quantified from scanned images and presented in the right panel. (**B**) EMSA was performed using Mock lysate (pET28) and *in vitro*-translated NsrR proteins (2 μM). C, probe only control. (**C**) Comparative effect of WT and V43A mutant NsrR expression on *hmp* transcription in *Salmonella*. The *hmp* null mutant *S.* Typhimurium strains harboring either pBAD18-*nsrR* or pBAD30-*nsrR*V43A, or both plasmids together, were cultured in EG media with or without GSNO (1 mM) to log-phase (OD_600_ = 0.5), followed by quantitative reverse transcription PCR (qRT-PCR) analysis. The relative *hmp* mRNA levels in strains cultured in GSNO-containing media compared with those without GSNO (UT) are shown as 2^−ΔΔCT^ value ± SD, analyzed from three independent experiments. The transcription of the housekeeping gene *rpoD* was used as a reference control. *P* values were determined by one-way analysis of variance. ***P < 0.001; ns, not significant.

To compare the binding affinity of purified His-tagged V43A mutant NsrR and WT NsrR proteins with DNA containing the NsrR binding site in the *hmp* promoter region, we performed the electrophoretic mobility shift assay (EMSA). In the absence of NO, as the concentration of both NsrR proteins increased, the amount of probe DNA bound by proteins appeared to increase proportionally (Fig. 2A). However, the detection of the shifted bands by the WT NsrR-DNA complex needed much longer exposure time compared with that of the V43A NsrR-DNA complex. This is evident from the varying intensities of unbound DNA probes in the two gels, demonstrating that the V43A mutant NsrR exhibited a more efficient binding to the DNA probe than the WT NsrR. This observation was further confirmed by EMSA with *in vitro*-translated NsrR proteins. The DNA probe showed a clear shift with the mutant NsrR under the same conditions where the WT NsrR did not produce a distinguishable shift (Fig. 2B). Additionally, the presence of super-shifted bands caused by an anti-His antibody confirmed the specificity of NsrR-DNA complexes. When proteins were exposed to NO before DNA binding, the intensities of shifted bands by the WT NsrR decreased, while those by the V43A NsrR remained unchanged (Fig. 2A). These results indicate that the V43A mutation increases the binding affinity of NsrR to the target DNA, even in the presence of NO.

To confirm the differential binding affinities of the WT NsrR and the V43A mutant NsrR to the *hmp* promoter in the cell, we transformed either the WT *nsrR* gene in a high-copy plasmid pBAD18 or the mutant *nsrR* V43A gene in a low-copy plasmid pBAD30 or both into WT and *hmp* null mutant *S.* Typhimurium. The *hmp* null mutant *Salmonella* still possesses the *hmp* promoter and downstream regions, allowing the measurement of *hmp* transcription by RT-PCR analysis. WT *Salmonella* harboring either the *nsrR* clone consistently repressed *hmp* transcription irrespective of NO exposure (data not shown), while the *hmp* null mutant *Salmonella* displayed different activities of two clones in a nitrosative stress-dependent manner (Fig. 2C). This suggests that the NO metabolism of the Hmp-expressing WT strain can minimize nitrosative stress required for the inactivation of NsrR encoded by the plasmids. In *hmp* mutant *S.* Typhimurium cultured in the presence of NO, the *nsrR*V43A clone repressed *hmp* transcription approximately sixfold more compared with the WT *nsrR* clone, and this level of repression was maintained in cells harboring both clones together. Considering the lower copy number of the *nsrR*V43A clone compared with the WT *nsrR* clone, these results demonstrate that the V43A mutant NsrR largely outcompetes the WT NsrR in binding to the *hmp* promoter, leading to significant inhibition of *hmp* transcription in *Salmonella* under nitrosative stress conditions. Collectively, these findings demonstrate that the V43A substitution mutant NsrR exhibits higher affinity binding to the *hmp* promoter than the WT NsrR, resulting in substantial inhibition of *hmp* transcription in *Salmonella* under nitrosative stress conditions.

### V43A mutation causes little effect to iron-sulfur cluster stability in NsrR

**Fig 3:**
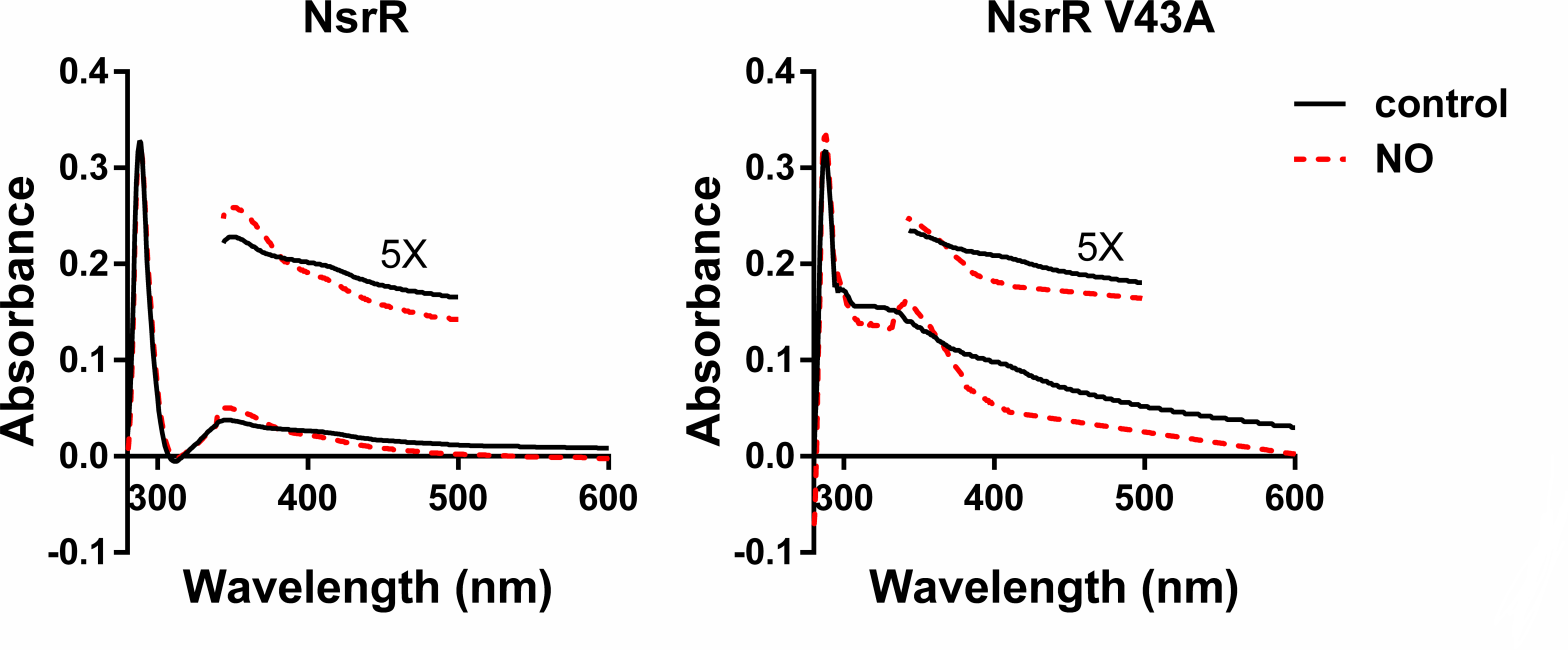
UV-visible spectroscopy of purified NsrR proteins. UV-visible spectra of purified WT and V43A NsrR (1 mg for each protein) in elution buffer (as described in Materials and Methods) before (solid line; control) and after Proli NONOate (100 μM) addition (dotted line; NO). The insets show magnified (×5) spectra from 340 to 500 nm wavelength.

Since the DNA binding affinity of NsrR is stabilized by the iron-sulfur cluster, the observed higher DNA-binding affinity of V43A NsrR, particularly when expressed via *in vitro* translation, may be due to the loss of the iron-sulfur cluster in NsrR, making it effective as an apo-form. To test this hypothesis, we performed UV visible spectroscopy of purified NsrR proteins (Fig. 3). The spectra of WT NsrR revealed broad weak bands with maximum intensity at 345 nm and a slight shoulder peak near 420 nm. When exposed to NO, the absorbance increased at 345 nm and decreased from 380 nm to longer wavelengths, compared with those without NO. These spectra, with and without NO treatment, were similar with those of [4Fe-4S] cluster-containing proteins FNR and WhiB, as well as ScNsrR (14, 17, 18), suggesting that StNsrR contains a [4Fe-4S] cluster. V43A NsrR exhibited similar spectra. Compared with WT NsrR, the overall absorbance levels were slightly higher, and NO treatment also induced a similar change pattern of increase at 345 nm and decrease in the visible wavelength region. This result suggests that the V43A mutation causes little effect on the stability of the iron-sulfur cluster in NsrR, allowing V43A NsrR to function as the holo-protein.

### Potential structural diversity in the DNA recognition helix α3 of NsrR proteins across bacterial orders

Studies that reported the first crystal structure of NsrR using *S. coelicolor* NsrR (ScNsrR) complexed with or without *hmpA1* promoter DNA have predicted that in the DNA recognition helix α3 of NsrR, the Ala44 residue of ScNsrR would be correspondent to Val43 of *Escherichia coli* NsrR (EcNsrR) or StNsrR and Glu43 of another NO-sensing Rrf2 family regulator IscR of *E. coli* (EcIscR) (16, 19).

**Fig 4:**
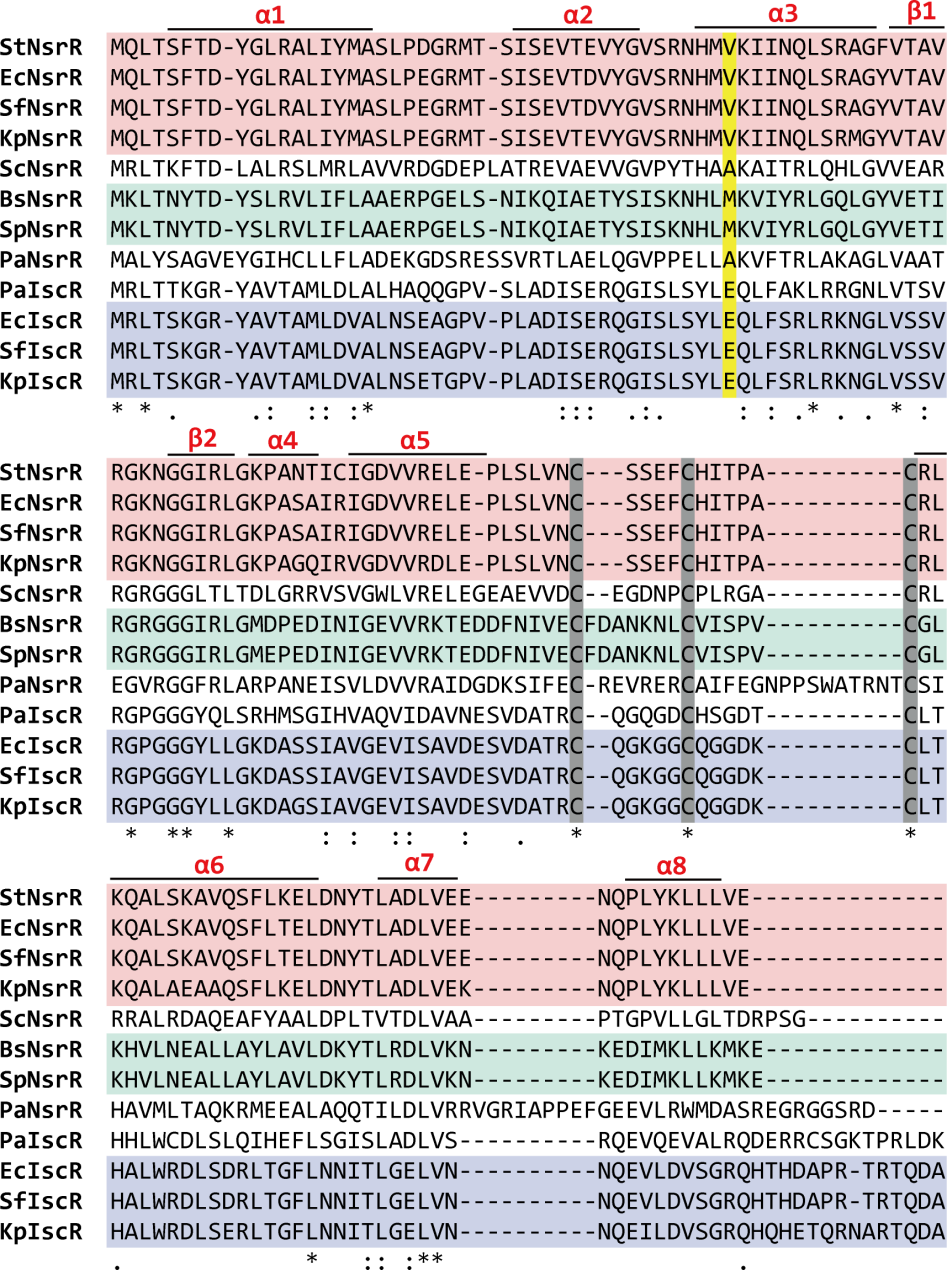
Comparison of NsrR and IscR protein amino acid sequences from various bacterial species. Sequence alignment was obtained by using the MUSCLE algorithm. Secondary structure elements (α helices and β sheets) are indicated based on *Streptomyces* NsrR structure. Conserved Cys residues are highlighted in gray, and the Val43 residue of enterobacterial NsrR and its corresponding amino acid residues in other bacteria and enterobacterial IscR are highlighted in yellow. The highly homologous sequences of enterobacterial NsrR, Bacillota NsrR, and enterobacterial IscR proteins are distinguished by highlighting them in pink, green, and purple, respectively. NsrR proteins included in the analysis are from *Salmonella* Typhimurium (StNsrR), *E. coli* (EcNsrR), *Shigella flexneri* (SfNsrR), *Klebsiella pneumonia* (KpNsrR), *Streptomyces coelicolor* (ScNsrR), *Bacillus subtilis* (BsNsrR), *Streptococcus pneumonia* (SpNsrR), and *Pseudomonas aeruginosa* (PaNsrR). IscR proteins included are from *P. aeruginosa* (PaIscR), *E. coli* (EcIscR), *S. flexneri* (SfIscR), and *K. pneumonia* (KpIscR). *, :, and . denote identical, highly conserved, and weakly conserved residues, respectively.

We performed a comparative analysis of the amino acid sequences of NsrR and IscR proteins from bacterial species selected from five different orders using the MUSCLE program (20) (Fig. 4). The analysis revealed conserved cysteine residues in all analyzed proteins, suggesting their potential involvement in Fe-S cluster assembly. Moreover, the N-terminal domain showed relatively higher sequence homology than the C-terminal domain, implying the presence of similar DNA binding domains in their structures. Enterobacterial NsrR proteins exhibited a strong sequence homology, indicating a conserved protein structure. In contrast, NsrR proteins from *S. coelicolor*, *B. subtilis*, *Streptococcus pneumonia*, and *Pseudomonas aeruginosa* displayed relatively diverse amino acid sequences compared with enterobacterial NsrR proteins. In the recognition helix α3, the strong homology of Val43 among enterobacterial NsrR proteins was not observed in NsrR from *S. coelicolor* (Ala44), *P. aeruginosa* (Ala45), *B. subtilis* (Met43), and *S. pneumonia* (Met43). All IscR proteins from *P. aeruginosa*, *Shigella flexneri*, *Klebsiella pneumonia*, and *E. coli* conserved the Glu43 residue. Given that the α3 helix serves as the DNA recognition helix in the defined structures of ScNsrR and EcIscR, our analysis suggests that the presence of diverse amino acids in the recognition helix α3 of NsrR proteins among bacterial orders may lead to potential variations in the recognition of target DNA during bacterial sensing of NO.

### The *nsrR*V43A and *nsrR*V43E mutations in *S.* Typhimurium genome inversely modulate *hmp* transcription and subsequent NO metabolism

**Fig 5:**
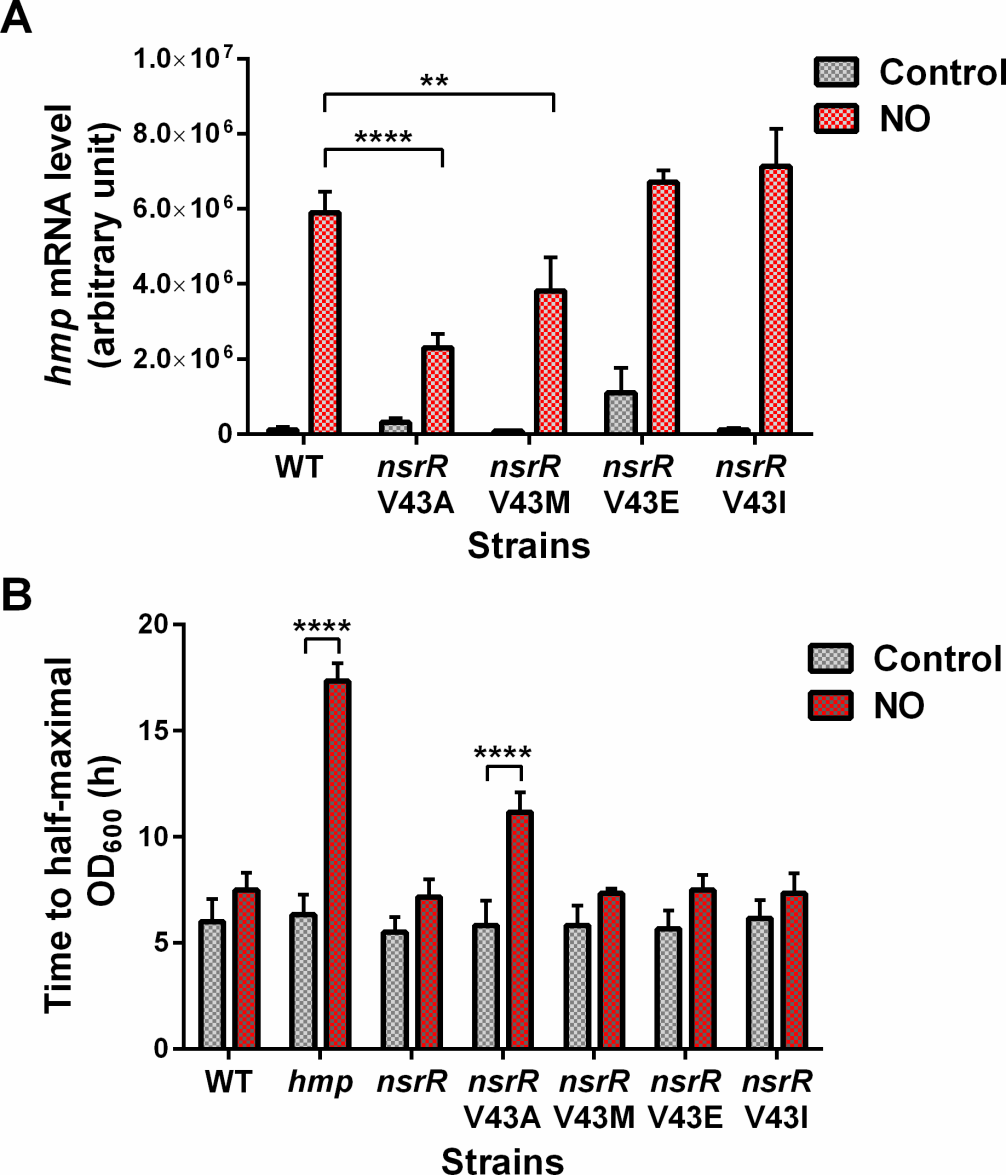
Effect of genomic mutations for substituting Val43 of NsrR on *S.* Typhimurium *hmp* transcription and growth in response to NO. (**A**) The effects of Val43 substitution mutations in the *S.* Typhimurium chromosome on *hmp* transcription was examined using culture conditions described in Fig. 2C. Values for *hmp* mRNA levels were normalized to *rpoD* mRNA levels. (**B**) Bacterial growth was assessed as described in Fig. 1B. **P  <  0.01 and ****P  <  0.0001, as determined by two-way analysis of variance.

**Fig 6:**
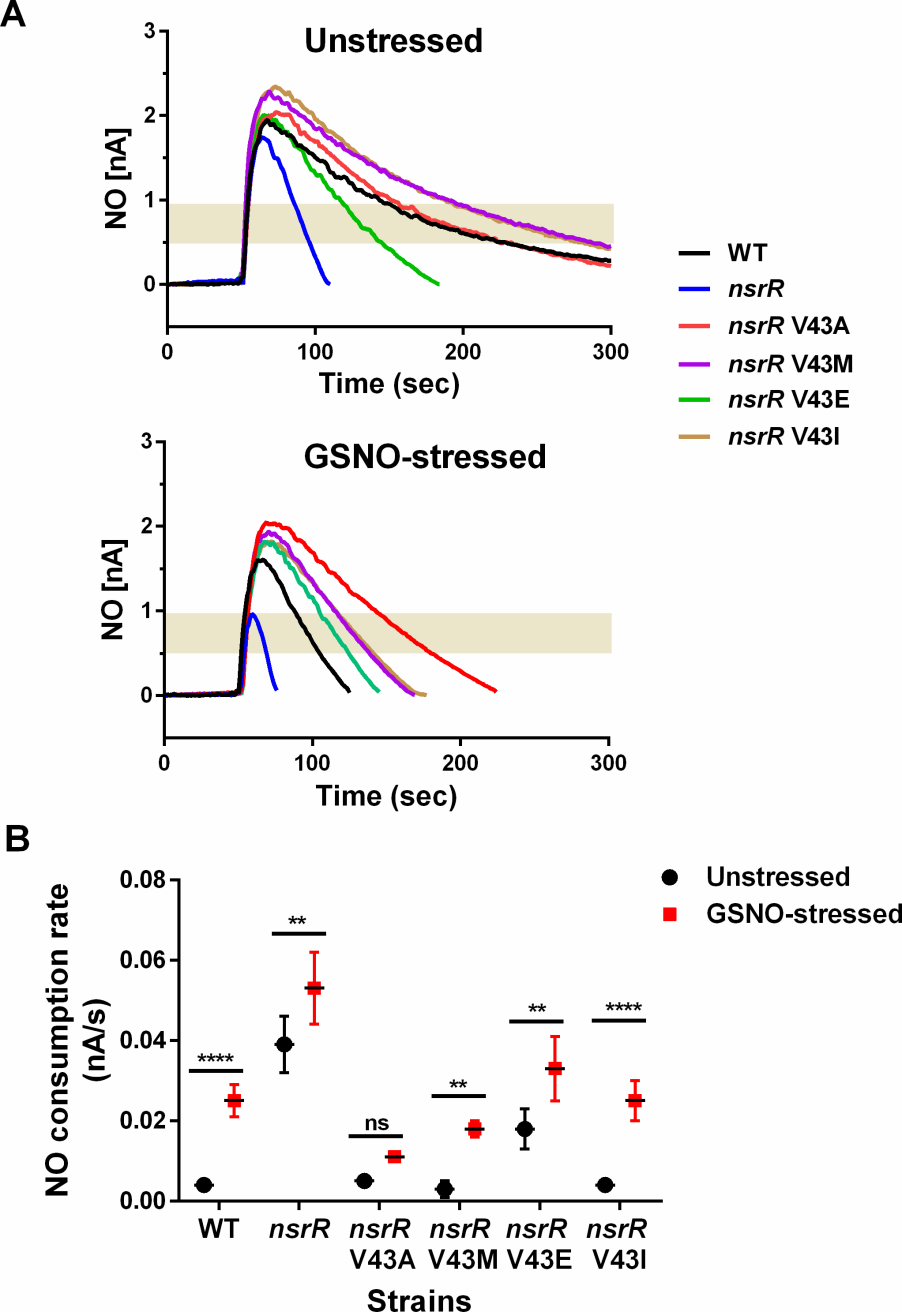
Effects of Val43 substitution mutations of NsrR on the NO consumption rate of *S.* Typhimurium. (**A**) *Salmonella* strains were cultured to mid log-phase (OD_600_ = 0.7), and samples were collected before (unstressed) and after treatment with GSNO (1 mM; GSNO-stressed) for 1 h. After adding Proli NONOate to each sample, the remaining concentration of NO was recorded for 5 min. (**B**) The rate of NO consumption was calculated based on the elapsed time for the change of NO concentration [from 0.8 nA to 0.5 nA; the gray area marked in (**A**)] measured in (**A**). Data are presented as the mean ± SD from three independent experiments. **P  <  0.01 and ****P  <  0.0001, as determined by two-way analysis of variance; ns, not significant.

To investigate the significance of the Val43 residue of StNsrR under more physiological conditions and also test the effects of substituting StNsrR Val43 with the corresponding amino acid residues found in NsrR and IscR proteins from other species as shown in Fig. 4, we performed the site-directed mutagenesis on the *nsrR* gene in the *S.* Typhimurium chromosome to express V43A, V43M, V43E, and V43I mutant NsrR in a single strain. Chromosomal *nsrR*V43A mutation substantially led to a severe reduction in *hmp* transcription and growth rate of *Salmonella* in NO-producing cultures compared with the WT and other *nsrR* mutations (Fig. 5), consistent with observations from *Salmonella* strains expressing V43A mutant NsrR from plasmids (Fig. 1). Conversely, the *nsrR*V43E mutant exhibited a significant (*P* < 0.001) increase in *hmp* transcription in cultures without NO, indicating a reduced binding affinity of this mutant NsrR to the *hmp* target DNA in *Salmonella*. V43M and V43I mutations had minimal effects on *hmp* transcription and bacterial growth rate. Next, we measured the NO consumption rate of *S.* Typhimurium expressing WT and mutant NsrR using the fast NO-releasing congener Proli NONOate before and after exposure to NO stress induced by GSNO (Fig. 6). The NO metabolic activity of *nsrR*V43A mutant did not show a significant increase with GSNO pretreatment compared with WT and other mutant *S.* Typhimurium strains that exhibited faster NO consumption. In the absence of GSNO pretreatment, only the *nsrR*V43E mutant showed a significant (*P* < 0.05) increase in NO consumption, reflecting the elevated *hmp* transcription in this mutant. WT, *nsrR*V43M, and *nsrR*V43I *S.* Typhimurium strains showed a similar increase in the NO consumption rate with GSNO pretreatment. These results demonstrate the importance of the Val43 residue in NsrR, particularly when substituted with Ala or Glu, in regulating *hmp* expression and subsequent NO metabolism in *Salmonella*.

## DISCUSSION

Our findings highlight the importance of Val43 residue in *Salmonella* NsrR for its interactions with the *hmp* target site and subsequent NO metabolism in *S.* Typhimurium, suggesting potential structural differences between enterobacterial NsrR and ScNsrR or IscR.

**Fig 7:**
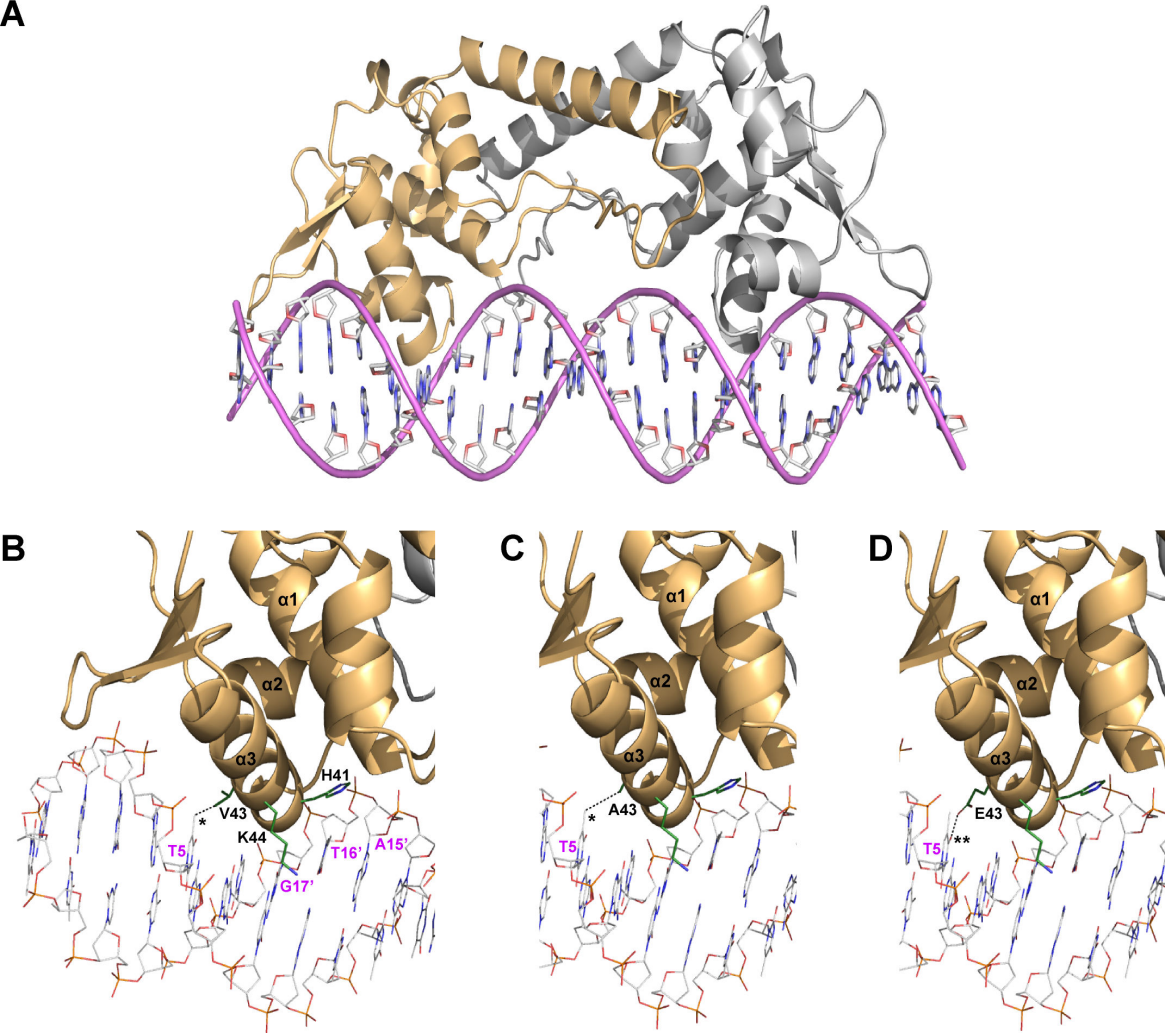
Structure models for WT and Val43 mutant NsrR proteins of *S.* Typhimurium interacting target DNA. (**A**) The 3D structure model of the homodimeric StNsrR bound to the target *hmp* DNA complex (StNsrR-*hmp*) was generated by superposing the algorithm-predicted StNsrR monomer and the DNA helix for NsrR target sequence on *hmp* with ScNsrR-*hmpA1* (blue) and EcIscR-*hya* (green) complexes using the PyMOL program. The model shown is the ribbon model of protein complexed with target DNA helix depicted as a stick model. NsrR monomers are colored gold and gray indicating α-helices numbered 1–3 for the N-terminal three helices and arrows denoting β-strands. The superposition result of the three protein-DNA complexes is shown in Fig. S4. (**B–D**) Close-up views of potential interactions between the α3 helices of WT (**B**), V43A (**C**), and V43E (**D**) NsrR proteins and the major groove of target *hmp* DNA are shown. The DNA helix bonds and NsrR residues that may interact with the DNA are depicted with sticks. * and ** denote the methyl group and carbonyl oxygen of thymine base (T5), respectively, which are in closest proximity to V43, A43, and E43 residues of StNsrR. In the DNA helices, complementary bases are denoted by adding an apostrophe (') next to the base position number.

Although we could not obtain a crystal structure of the StNsrR-*hmp* complex, the use of advanced algorithms, such as AlphaFold and Avogadro (21, 22), allowed us to predict a 3D complex structure. The predicted StNsrR structure exhibited the characteristic winged helix-turn-helix (wHTH) protein structure of the Rrf2 family, similar to the ScNsrR and EcIscR proteins when superposed together (Fig. 7A; Fig. S4). Comparisons between the algorithm-predicted StNsrR-*hmp* complex structure and the known structures of ScNsrR-*hmpA1* and EcIscR-*hya* complexes revealed potential similarities and differences in the spatial stoichiometry of α3 helices and their interacting target DNAs (Fig. S4 and S5). The imidazole ring of StNsrR His41 residue that is conserved in NsrR proteins of most bacteria examined in this study (Fig. 4) likely interacts with the phosphate backbone between thymine (T16') and adenine (A15') bases, similar to the case of ScNsrR His42 residue (19) (Fig. 7B). Lys44 may also assist NsrR in recognition of target DNA by possibly interacting with guanine (G17') or thymine (T16') bases. Val43 of StNsrR is expected to correspond to Ala44 of ScNsrR and Glu43 of EcIscR, as suggested by structural studies of ScNsrR and by our MUSCLE analysis (16, 19) (Fig. 4). Although Ala44 of ScNsrR does not interact with bases (19), the methyl side chain of Val43 in the StNsrR-*hmp* complex model is in close proximity (2.4 Å) to the C7 methyl group of the thymine base (T5) (Fig. 7B). Conversely, the substitution of Val43 with Ala results in a longer distance (3.9 Å) to this methyl group, which may enable stable hydrophobic interactions between them (Fig. 7C). This change in distance between the methyl side chains of Val and Ala and the methyl group of thymine provides a potential explanation for why the NsrRV43A mutant protein strongly interacts with the target *hmp* DNA regardless of NO exposure. The fact that the purified V43A NsrR protein still retains its iron-sulfur cluster, which remains susceptible to NO modification, further supports this hypothesis. It also suggests that the closer proximity of WT NsrR Val43 residue may lead to collision and subsequent dissociation of NsrR from the target DNA due to subtle changes in NsrR conformation induced by NO-mediated iron-sulfur cluster modification.

In contrast, the substitution of Val43 with Glu may result in electrostatic repulsion between the carboxyl side chain of Glu43 and the carbonyl oxygen of the thymine base (Fig. 7D), potentially leading to constitutive *hmp* expression and NO consumption in *Salmonella* expressing the NsrRV43E variant without prior NO treatment. In fact, Glu43 of EcIscR allows IscR to discriminate between two different DNA binding sites (type 1 and type 2 sites) in regulating the Isc Fe-S biogenesis pathway. In the structure of apo-EcIscR bound to the target DNA in the *hya* promoter, Glu43 is critical for selectively binding to C7C8 and C7'A8' bases in the type 2 site by forming a bidentate interaction with the exocyclic amines of bases. However, it does not interact with T7'T8' bases in the type 1 site due to inhibitory interactions with thymine bases (23). Similarly, it can be assumed that T5 and T5' bases in the NsrR target sequence in the *Salmonella hmp* promoter may experience repulsive interactions with the carboxyl side chain of the Glu43 residue of each NsrR monomers. Considering the NsrR DNA binding sites of *E. coli* and *Salmonella*, which consist of 23-bp regions composed of inverted duplicates of an 11-bp sequence (AAGATGTATTT) separated by a variable base (7, 24, 25) (Fig. S5), the highly conserved fifth thymine base appears to be critically important for discriminating the α3 helix of enterobacterial NsrR from those of NsrR proteins in bacteria belonging to a different order. Notably, *S. coelicolor hmpA1* has cytosine at this base position, which lacks a methyl group that could otherwise interact with Ala44 of ScNsrR. These comparisons suggest that the heterogeneity in amino acid residues in the recognition helix (α3) would aid structurally related Rrf2 family response regulators in recognizing bona fide target sites.

Initially, this study aimed to determine the molecular structure of both WT NsrR and a mutant NsrR capable of binding target genes regardless of NO exposure. A comparative analysis of these structures would have provided valuable insights into the molecular pathway by which NsrR senses NO in enterobacteria. Although we encountered challenges in crystallizing both NsrR proteins, our study identified Val43 in StNsrR as a crucial amino acid for regulating *hmp* transcription in *Salmonella*, particularly in response to and detoxifying NO. The high homology of NsrR amino acid sequences in enterobacteria, distinct from those in NsrR proteins found in other bacterial orders, underscores the significance of our observations in understanding bacterial NO responses in enteric pathogens that must cope with NO stress in animal hosts.

## MATERIALS AND METHODS

### Bacterial strains and culture conditions

*Salmonella enterica* serovar Typhimurium 14028S was used for WT parental strain, and its isogenic gene mutants were used for this study and listed in Table S1. *E. coli* BL21(DE3) was utilized to express and purify NsrR proteins. All bacterial strains were cultured at 37℃ unless otherwise specified. Construction of gene mutations in *S.* typhimurium was carried out using the PCR-mediated one-step gene mutation method with λ Red recombinase (26). Subsequent recombination processes of gene mutation, including transduction to the fresh WT strain and removal of antibiotic cassettes in mutants, were performed as described previously (27). The DNA primers used for these procedures are listed in Table S2. All mutants and plasmids constructed in this study were verified by DNA sequencing (Macrogen Inc.). For routine bacterial cultures, Luria-Bertani (LB) complex (Difco) or minimal E glucose (0.2%) media (28) were used. Antibiotics were added at the following concentrations to LB media when required: kanamycin (50 μg mL^−1^), ampicillin (100 μg mL^−1^), or chloramphenicol (20 μg mL^−1^). For NO congeners, spermine NONOate and Proli NONOate, purchased from Cayman chemical, and S-nitrosoglutathione, synthesized through the reaction of glutathione and acidified sodium nitrite, were used in this study (29). All chemicals were purchased from Sigma-Aldrich unless otherwise stated.

### Random mutagenesis of NsrR

To introduce random mutations in the *nsrR* gene, an *nsrR* clone plasmid previously constructed (3) was used as the template plasmid for PCR. Mutagenic PCR was performed using the pBAD-Fw and pBAD-Rev primers in a reaction buffer of 7 mM MgCl_2_, 50 mM KCl, 10 mM Tris-HCl, pH 8.3, 0.5 mM MnCl_2_, 0.01% gelatin, and varying concentrations of the four dNTPs (2 mM dATP, 10 mM dCTP, 2 mM dGTP, and 10 mM dTTP), as described in reference (30). The purified PCR products were then digested with EcoRI and HindIII restriction enzymes and ligated into the pBAD30 plasmid digested with the same enzymes. The ligation mixtures were electroporated into the IB1076 (*hmp-lacZ*), and colonies displaying distinguishable changes of LacZ activity were selected after culture on the solid EG media containing X-Gal (25 μg mL^−1^) and GSNO (250 μM). DNA sequences of mutant *nsrR* clones that exhibited regulatory effects on *hmp* transcription were analyzed.

### Site-directed mutation of *nsrR*

Site-directed mutation of *nsrR* on the plasmid (pBAD-*nsrR*) was conducted using a two-step PCR approach with DNA primers containing the desired base changes. To construct specific base mutations in the *nsrR* gene, two PCR products were generated from the first PCR reactions using primer pairs of pBAD-Fw and “primer for mutation”-Rev and “primer for mutation”-Fw and pBAD-Rev, respectively. These two PCR products were mixed, heat-denatured for 10 min at 95°C, and slowly annealed at room temperature. The resulting mixture was used as the template for second PCR using primers pBAD-Fw and pBAD-Rev. The purified PCR products were cloned into pBAD30 using the same enzymes described in the random mutagenesis methods. To introduce site-directed mutation of *nsrR* in the *S.* Typhimurium chromosome, we employed a combinational recombination method utilizing the Red recombination system and I-*Sce*I endonuclease (31). The desired mutation site was first recombined with Cm^R^ cassette and I-SceI recognition site using the Red recombinase. The DNA product amplified from pWRG100 with the primers listed in Table S2 was used for this recombination. Subsequently, the cassette was replaced with the desired mutated bases using the I-SceI endonuclease encoded by pWRG99 and the 80mer-annealed dsDNA containing the specific base changes.

### Measurement of *hmp* transcription

To measure *hmp* transcription, two complementary methods were used. For the *hmp-lacZ* transcriptional fusion, β-galactosidase activity was measured following the method described in reference (32). To measure *hmp* mRNA levels, qRT-PCR was performed as reported previously (33). Briefly, for measuring β-galactosidase activity, *S.* Typhimurium strains grown overnight in LB broth were diluted at 1:200 (vol/vol) in EG media with or without GSNO (500 μM) and grown to early log phase (OD_600_ = 0.5). For qRT-PCR, log-phase cultures grown as mentioned above but without GSNO were divided, and half of the culture was treated with GSNO (1 mM) and further cultured for 1 h. Bacterial transcription was stopped by mixing a one-fifth volume of ice-cold phenol/ethanol (5% phenol in 95% ethanol) solution with the culture before harvesting the cells. Total RNA was extracted using RNAiso Plus reagent (Takara Bio) and subjected to qRT-PCR analysis using a QuantiTect SYBR Green RT-PCR Kit (Qiagen) with primer pairs *hmp*-Fw and *hmp*-Rev.

### Measurement of nitric oxide susceptibility

*Salmonella* strains were grown overnight in LB broth and then diluted in phosphate-buffered saline (PBS) to an OD_600_ of 1. An equal amount of the diluted culture (OD_600_ = 0.02) was inoculated into EG media supplemented with or without GSNO (500 μM). The growth kinetics of the strains was assessed using the BioScreen C Microbiology Microplate Reader (Labsystems), measuring OD_600_ every 30 min for 24 h at 37°C with agitation.

### Expression and purification of WT and V43A mutant NsrR and UV-visible spectroscopy

To express and purify WT and V43A mutant NsrR proteins, we constructed pET28a plasmid-based clones containing the WT *nsrR* and the *nsrR*V43A genes, which were PCR amplified from chromosomal DNA and pBAD30-*nsrR*V43A, respectively. These N-terminal His-tagged proteins were expressed by culturing the log-phase (OD_600_ ~0.5) cells of *E. coli* BL21 harboring the clones in LB broth supplemented with IPTG (0.5 mM) for 16 h at 18°C. The harvested cells were then resuspended in 50-mL lysis buffer containing 20 mM Tris-HCl, pH7.5, 500 mM NaCl, 5 mM MgCl_2_, 10 mM imidazole, 1 mM EDTA, 1 mM DTT, 1 mM PMSF, and DNase I. The resuspended cells were sonicated (two 10-sec pulses at 25% amplitude with a 2-min rest on ice) and centrifuged (2,000 × *g*). The filtered supernatant (0.45 μm pore) was passed through a column containing Ni-NTA agarose resins (Qiagen) using an Econo pump (0.5 mL min^−1^). After protein binding, Ni-NTA resins were washed with 2 mL washing buffer (20 mM Tris-HCl, pH7.5, 20 mM imidazole, 500 mM NaCl, and 1 mM DTT) for 1 min, and the His-tagged proteins were eluted with elution buffer (20 mM Tris-HCl, pH 7.5, 500 mM imidazole, and 500 mM NaCl). The purified His-tagged proteins were dialyzed overnight at 4°C in a buffer (20 mM Tris-HCl, pH 7.5, 200 mM NaCl, 0.5 mM EDTA, 10% glycerol, and 1 mM DTT) using a dialysis membrane (MWCO 10KD; Spectrum Laboratories Inc.). When challenging the protein crystallization process, the His-tag was removed by digestion of the purified proteins with human α-thrombin (7 unit, 10,000× dilution in 2 mM CaCl_2_) at room temperature for 6 h. After re-subjection of the digestion mixture onto the Ni-affinity column, the digested proteins in the flow-through fractions were concentrated and finally isolated by size-exclusion chromatography using a HiLoad 16/600 Superdex 200 pg column (GE Healthcare) in the buffer (20 mM Tris-HCl, pH 7.5, and 50 mM NaCl). For *in vitro* synthesis of His-tagged NsrR proteins, the TNT T7 Quick Coupled Transcription/Translation System (Promega) was used according to the provided protocols. One microgram of the pET28a-*nsrR* or pET28a-*nsrR*V43A clone was added to the TNT Quick Master Mix and incubated for 1 h at 30°C. We used UV-visible absorbance spectroscopy to determine the presence of a iron-sulfur cluster in purified NsrR proteins. The spectra were acquired using a Hitachi U-1900 spectrophotometer at room temperature. The UV-visible spectra of 1-mL NsrR samples in elution buffer were measured from 280 to 600 nm before and after treatment with a fast-releasing NO donor Proli NONOate (100 μM, Cayman Chemical). The spectra were recorded and analyzed using UV Solutions software (Hitachi).

### Electrophoretic mobility shift assay

EMSA reactions were conducted under ambient aerobic conditions in a protein-DNA binding buffer (20 mM Tris-HCl, pH 7.5, 200 mM NaCl, 10 mM MgCl_2_, 0.5 mM EDTA, 10% glycerol, and 1 mM DTT). The resulting products were resolved on polyacrylamide gel electrophoresis using 5% native gels in 0.5% TBE (Tris-borate-EDTA) buffer (Welgene). The dsDNA *hmp* probes were generated by annealing the reverse-complementary oligonucleotides (Table S2), encompassing the 19-bp NsrR binding consensus sequence conserved in γ-proteobacteria (7). The probes were then labeled with [γ-^32^P]-ATP by T4 polynucleotide kinase (Promega). For all EMSA experiments, 0.1 ng of *hmp* DNA probe was incubated with NsrR proteins at fourfold increasing concentrations (from 62.5 pM to 4 μM) for 15 min. In super shift assays, an anti-His antibody (0.2 μg, Santacruz) was added to the reactions. Intensities of the bands in gel images were quantified using the NIH ImageJ software.

### Measurement of NO consumption rate

The NO-consuming ability of *Salmonella* strains was analyzed following the procedure described in the previous report (27). Briefly, mid log-phase cells (OD_600_ = 0.7), treated with or without GSNO (1 mM) for 1 h, were harvested and washed with PBS and resuspended in 10 mL pre-warmed PBS. Cell suspensions were transferred into a multiport measurement chamber (NOCHM-4; WPI Inc.) equipped with an ISO-NOP NO sensor (WPI Inc.) set at 37°C, which was connected to a free radical analyzer (TBR4100; WPI Inc.). The NO consumption rate was determined by measuring the remaining NO in the buffer after adding Proli NONOate (2 μM). The data signal was recorded using the LabChart program (WPI Inc.).

### Modeling of the StNsrR-*hmp* complex structure

To generate an StNsrR-*hmp* complex structure model, we used the monomer structure of *S.* Typhimurium NsrR (UniProt ID Q8ZKA3) predicted by AlphaFold (21), a recently developed deep-learning molecular modeling program. The B-DNA helix model of the NsrR target sequence (5′-AAGATGCATTTGATATACATCAT-3′) on the *S.* Typhimurium *hmp* promoter was generated using the Avogadro program (22). Then, the predicted StNsrR and *hmp* DNA structures were superposed with defined protein-DNA complex structures of ScNsrR-*hmpA1* (PDB ID 7B0C) and EcIscR-*hya* (PDB ID 4HF1) in the PyMOL program (The PyMOL Molecular Graphics System, version 2.0 Schrödinger, LLC). The resultant predicted structure of dimeric StNsrR and *hmp* DNA complex was used for assessing potential interactions between WT and V43 variants of StNsrR proteins and target DNA, including spatial stoichiometry.

### Statistical analysis

Statistical analyses were performed using the program GraphPad Prism (version 6; GraphPad Software, USA)

## SUPPLEMENTAL MATERIAL

Supplemental tables and figures spectrum.03024-23-s0001.pdf Supplementary information.

ASM does not own the copyrights to Supplemental Material that may be linked to, or accessed through, an article. The authors have granted ASM a non-exclusive, world-wide license to publish the Supplemental Material files. Please contact the corresponding author directly for reuse.

## References

[1] Fang FC , Vázquez-Torres A . 2019. Reactive nitrogen species in host-bacterial interactions. Curr Opin Immunol 60:96–102. doi:10.1016/j.coi.2019.05.008 31200187 PMC6800629

[2] Ruby T , McLaughlin L , Gopinath S , Monack D . 2012. Salmonella’s long-term relationship with its host. FEMS Microbiol Rev 36:600–615. doi:10.1111/j.1574-6976.2012.00332.x 22335190

[3] Bang IS , Liu L , Vazquez-Torres A , Crouch ML , Stamler JS , Fang FC . 2006. Maintenance of nitric oxide and redox homeostasis by the Salmonella flavohemoglobin Hmp. J Biol Chem 281:28039–28047. doi:10.1074/jbc.M605174200 16873371

[4] Poole RK . 2020. Flavohaemoglobin: the pre-eminent nitric oxide-detoxifying machine of microorganisms. F1000Res 9:F1000 Faculty Rev-7. doi:10.12688/f1000research.20563.1 PMC6950321 31956400

[5] Forrester MT , Foster MW . 2012. Protection from nitrosative stress: a central role for microbial flavohemoglobin. Free Radic Biol Med 52:1620–1633. doi:10.1016/j.freeradbiomed.2012.01.028 22343413

[6] Hausladen A , Stamler JS . 2012. Is the flavohemoglobin a nitric oxide dioxygenase? Free Radic Biol Med 53:1209–1210. doi:10.1016/j.freeradbiomed.2012.06.033 22796210

[7] Rodionov DA , Dubchak IL , Arkin AP , Alm EJ , Gelfand MS . 2005. Dissimilatory metabolism of nitrogen oxides in bacteria: comparative reconstruction of transcriptional networks. PLoS Comput Biol 1:e55. doi:10.1371/journal.pcbi.0010055 16261196 PMC1274295

[8] Sevilla E , Bes MT , González A , Peleato ML , Fillat MF . 2019. Redox-based transcriptional regulation in prokaryotes: revisiting model mechanisms. Antioxid Redox Signal 30:1651–1696. doi:10.1089/ars.2017.7442 30073850

[9] McLean S , Bowman LAH , Poole RK . 2010. Peroxynitrite stress is exacerbated by flavohaemoglobin-derived oxidative stress in Salmonella Typhimurium and is relieved by nitric oxide. Microbiology (Reading) 156:3556–3565. doi:10.1099/mic.0.044214-0 20829289

[10] Gilberthorpe NJ , Lee ME , Stevanin TM , Read RC , Poole RK . 2007. NsrR: a key regulator circumventing Salmonella enterica serovar Typhimurium oxidative and nitrosative stress in vitro and in IFN-gamma-stimulated J774.2 macrophages. Microbiology (Reading) 153:1756–1771. doi:10.1099/mic.0.2006/003731-0 17526833 PMC2884951

[11] Tucker NP , Le Brun NE , Dixon R , Hutchings MI . 2010. There’s NO stopping NsrR, a global regulator of the bacterial NO stress response. Trends Microbiol 18:149–156. doi:10.1016/j.tim.2009.12.009 20167493

[12] Kommineni S , Yukl E , Hayashi T , Delepine J , Geng H , Moënne-Loccoz P , Nakano MM . 2010. Nitric oxide-sensitive and -insensitive interaction of Bacillus subtilis NsrR with a ResDE-controlled promoter. Mol Microbiol 78:1280–1293. doi:10.1111/j.1365-2958.2010.07407.x 21091510 PMC3075490

[13] Isabella VM , Lapek JD , Kennedy EM , Clark VL . 2009. Functional analysis of NsrR, a nitric oxide-sensing Rrf2 repressor in Neisseria gonorrhoeae. Mol Microbiol 71:227–239. doi:10.1111/j.1365-2958.2008.06522.x 19007408 PMC2630374

[14] Crack JC , Munnoch J , Dodd EL , Knowles F , Al Bassam MM , Kamali S , Holland AA , Cramer SP , Hamilton CJ , Johnson MK , Thomson AJ , Hutchings MI , Le Brun NE . 2015. NsrR from Streptomyces coelicolor is a nitric oxide- sensing [4Fe-4S] cluster protein with a specialized regulatory function. J Biol Chem 290:12689–12704. doi:10.1074/jbc.M115.643072 25771538 PMC4432287

[15] Yukl ET , Elbaz MA , Nakano MM , Moënne-Loccoz P . 2008. Transcription factor NsrR from Bacillus subtilis senses nitric oxide with a 4Fe-4S cluster (†). Biochemistry 47:13084–13092. doi:10.1021/bi801342x 19006327 PMC2891187

[16] Volbeda A , Dodd EL , Darnault C , Crack JC , Renoux O , Hutchings MI , Le Brun NE , Fontecilla-Camps JC . 2017. Crystal structures of the NO sensor NsrR reveal how its iron-sulfur cluster modulates DNA binding. Nat Commun 8:15052. doi:10.1038/ncomms15052 28425466 PMC5411485

[17] Crack JC , Stapleton MR , Green J , Thomson AJ , Le Brun NE . 2013. Mechanism of [4Fe-4S](Cys)4 cluster nitrosylation is conserved among NO-responsive regulators. J Biol Chem 288:11492–11502. doi:10.1074/jbc.M112.439901 23471974 PMC3630887

[18] Crack JC , Smith LJ , Stapleton MR , Peck J , Watmough NJ , Buttner MJ , Buxton RS , Green J , Oganesyan VS , Thomson AJ , Le Brun NE . 2011. Mechanistic insight into the nitrosylation of the [4Fe-4S] cluster of WhiB-like proteins. J Am Chem Soc 133:1112–1121. doi:10.1021/ja109581t 21182249 PMC3117330

[19] Rohac R , Crack JC , de Rosny E , Gigarel O , Le Brun NE , Fontecilla-Camps JC , Volbeda A . 2022. Structural determinants of DNA recognition by the NO sensor NsrR and related Rrf2-type [FeS]-transcription factors. Commun Biol 5:769. doi:10.1038/s42003-022-03745-7 35908109 PMC9338935

[20] Edgar RC . 2004. MUSCLE: multiple sequence alignment with high accuracy and high throughput. Nucleic Acids Res 32:1792–1797. doi:10.1093/nar/gkh340 15034147 PMC390337

[21] Jumper J , Evans R , Pritzel A , Green T , Figurnov M , Ronneberger O , Tunyasuvunakool K , Bates R , Žídek A , Potapenko A , et al. . 2021. Highly accurate protein structure prediction with AlphaFold. Nature 596:583–589. doi:10.1038/s41586-021-03819-2 34265844 PMC8371605

[22] Hanwell MD , Curtis DE , Lonie DC , Vandermeersch T , Zurek E , Hutchison GR . 2012. Avogadro: an advanced semantic chemical editor, visualization, and analysis platform. J Cheminform 4:17. doi:10.1186/1758-2946-4-17 22889332 PMC3542060

[23] Rajagopalan S , Teter SJ , Zwart PH , Brennan RG , Phillips KJ , Kiley PJ . 2013. Studies of IscR reveal a unique mechanism for metal-dependent regulation of DNA binding specificity. Nat Struct Mol Biol 20:740–747. doi:10.1038/nsmb.2568 23644595 PMC3676455

[24] Partridge JD , Bodenmiller DM , Humphrys MS , Spiro S . 2009. NsrR targets in the Escherichia coli genome: new insights into DNA sequence requirements for binding and a role for NsrR in the regulation of motility. Mol Microbiol 73:680–694. doi:10.1111/j.1365-2958.2009.06799.x 19656291

[25] Karlinsey JE , Bang IS , Becker LA , Frawley ER , Porwollik S , Robbins HF , Thomas VC , Urbano R , McClelland M , Fang FC . 2012. The NsrR regulon in nitrosative stress resistance of Salmonella enterica serovar Typhimurium. Mol Microbiol 85:1179–1193. doi:10.1111/j.1365-2958.2012.08167.x 22831173 PMC3438343

[26] Datsenko KA , Wanner BL . 2000. One-step inactivation of chromosomal genes in Escherichia coli K-12 using PCR products. Proc Natl Acad Sci U S A 97:6640–6645. doi:10.1073/pnas.120163297 10829079 PMC18686

[27] Park YM , Lee HJ , Jeong J-H , Kook J-K , Choy HE , Hahn T-W , Bang IS . 2015. Branched-chain amino acid supplementation promotes aerobic growth of Salmonella Typhimurium under nitrosative stress conditions. Arch Microbiol 197:1117–1127. doi:10.1007/s00203-015-1151-y 26374245

[28] Vogel HJ , Bonner DM . 1956. Acetylornithinase of Escherichia coli: partial purification and some properties. J Biol Chem 218:97–106. 13278318

[29] Hart TW . 1985. Some observations concerning the S-nitroso and S-phenylsulphonyl derivatives of L-cysteine and glutathione. Tetrahedron Lett 26:2013–2016. doi:10.1016/S0040-4039(00)98368-0

[30] Cadwell RC , Joyce GF . 1992. Randomization of genes by PCR mutagenesis. PCR Methods Appl 2:28–33. doi:10.1101/gr.2.1.28 1490172

[31] Blank K , Hensel M , Gerlach RG . 2011. Rapid and highly efficient method for scarless mutagenesis within the Salmonella enterica chromosome. PLoS One 6:e15763. doi:10.1371/journal.pone.0015763 21264289 PMC3021506

[32] Maloy S . 1990. Experimental techniques in bacterial genetics. Jones and Bartlett Publishers, Boston, MA.

[33] Cho Y , Park YM , Barate AK , Park SY , Park HJ , Lee MR , Truong QL , Yoon JW , Bang IS , Hahn TW . 2015. The role of rpoS, Hmp, and ssrAB in Salmonella enterica gallinarum and evaluation of a triple-deletion mutant as a live vaccine candidate in lohmann layer chickens. J Vet Sci 16:187–194. doi:10.4142/jvs.2015.16.2.187 25549217 PMC4483502

