# Supplementary material for: Val43 residue of NsrR is crucial for the nitric oxide response of Salmonella Typhimurium
Source: Microbiol Spectr. 2023 Dec 6;12(1):e03024-23. doi: 10.1128/spectrum.03024-23 (PMC10783083; doi:10.1128/spectrum.03024-23)
Supplement: Supplemental tables and figures — Supplementary information. [file spectrum.03024-23-s0001.pdf]

Supplementary information for;

Val43 residue of NsrR is crucial for nitric oxide response of *Salmonella* Typhimurium

Hee Jeong Park[1], Hye Won Jeong[1], Choa Lee[1], Mi Rae Lee[1], Hojung Choi[2], Eungseok Kim[2], and Iel Soo Bang[1]#

[1] Department of Microbiology and Immunology, Chosun University School of Dentistry, Gwangju 61452, [2] Department of Biological Sciences, College of Natural Sciences, Chonnam National University, Gwangju 61186, Republic of Korea

# Corresponding Author: isbang@chosun.ac.kr

This supplementary information includes;

Table S1. Bacterial strains and plasmids used in this study.

Table S2. Oligonucleotides used in this study.

Figure S1. Screening for the NsrR mutant clones that alter the *hmp-lacZ* expression.

Figure S2. The *hmp-lacZ* expression of the *nsrR* mutant clones selected through random mutagenesis.

Figure S3. The effect of *nsrR* mutant clones on the *S.* Typhimurium growth.

Figure S4. Superposition of the predicted StNsrR-*hmp* complex structure with ScNsrR-*hmpA1* and EcIscR-*hya* complex structures.

Figure S5. NsrR binding consensus DNA sequences of *S. coelicolor* and *S.* Typhmurium.

Table S1. Bacterial strains and plasmids used in this study

Strains	Genotype	Source or Reference
IB1	*S.* Typhimurium 14028s, Wild-type	ATCC
IB3	*hmp*::KM	(1)
IB1231	*hmp-lacZ*::KM	(2)
IB685	*ΔnsrR*::FRT	(1)
IB696	*ΔnsrR*::FRT /pBAD30	(1)
IB697	*ΔnsrR*::FRT /pBAD30-*nsrR*	(1)
IB1230	*ΔnsrR*::FRT *hmp-lacZ*::KM	This study
IB1750	*ΔnsrR*::FRT *hmp-lacZ*::KM /pBAD30	
IB2106	*ΔnsrR*::FRT *hmp-lacZ*::KM /pBAD30-*nsrR*	This study
IB2108	*ΔnsrR*::FRT *hmp-lacZ*::KM /pBAD30-*nsrR* (mutant B4; M17L and V43A*)*	This study
IB2088	*ΔnsrR*::FRT *hmp-lacZ* ::KM /pBAD30-*nsrR* M17L	This study
IB2089	*ΔnsrR*::FRT *hmp-lacZ* ::KM /pBAD30-*nsrR* V43A	This study
IB2087	*hmp-lacZ*::KM /pBAD30-*nsrR* V43A	This study
IB2603	*hmp-lacZ*::KM /pBAD18-*nsrR*	This study
IB2604	*hmp-lacZ*::KM /pBAD18-*nsrR /*pBAD30-*nsrR* V43A	This study
IB1646	*nsrR* V43A	This study
IB2305	*nsrR* V43M	This study
IB2306	*nsrR* V43E	This study
IB2307	*nsrR* V43I	This study
BL21	*Escherichia coli* F^- *ompT hsdSB* (rB^- mB^-) *gal dcm* (DE3)	Sigma-Aldrich
IB1507	BL21 /pET28a(+)-*nsrR*	This study
IB1609	BL21 /pET28a(+)-*nsrR* V43A	This study
**Plasmids**		
pKD3	Plasmid carrying the FRT-Cm^r-FRT-cassette	(3)
pKD4	Plasmid carrying the FRT-Km^r-FRT-cassette	(3)
pKD46	Plasmid encoding arabinose-inducible lamda red recombinase	(3)
pTP233	Plasmid encoding IPTG-inducible lamda red recombinase	(4)

pCP20	Plasmid expressing the FLP recombinase	(5)
pBAD18	Gene expression vector	(6)
pBAD30	Gene expression vector	(6)
pET28a	Gene expression vector	Novagen
pWRG99	AHT-inducible I-*Sce*I endonuclease	(7)
pWRG100	Plasmid carrying CM^r cassette and an I-*Sce*I recognition site	(7)

Table S2. Oligonucleotides used in this study

	Sequence (5′ → 3′)[*]
Recombinant plasmid construction	
pBAD-Fw	TCGCAACTCTCTACTGTTTC
pBAD-Rev	GCCTGATACAGATTAAATCAG
nsrR M17L-Fw	CTAATCTATTTGGCGTCGTTA
nsrR M17L-Rev	TAACGACGCCAAATAGATTAG
nsrR V43A-Fw	TAATCATATGGCCA AAATTATCA
nsrR V43A-Rev	TGATAATTTTGGCCATATGATTA
Real time RT-PCR	
rpoD-Fw	GTGAAATGGGCACTGTTGAACTG
rpoD-Rev	TTCCAGCAGATAGGTAATGGCTTC
hmp-Fw	CGCTACAGTAAAGGCCACCA
hmp-Rev	CCCGACGATGTTGTACTGCT
DNA probe for EMSA	
NsrR-*hmp*-FW	AAGATGCATTTGATATACATCATT
NsrR-*hmp*-Rev	AATGATGTATATCAAATGCATCTT
Chromosomal mutation	
nsrR-I-SceI-cat-Fw	GTGACAGAAGTCTACGGCGTGTCCCGTAATCATATGGCCAC GCCTTACGCCCCGCCCTGC
nsrR-I-SceI-cat-Rev	GCGGTGACAAAGCCCGCCCGGCTAAGCTGATTGATAATTTC TAGACTATATTACCCTGTT
nsrR V43A-Fw	GTGACAGAAGTCTACGGCGTGTCCCGTAATCATATGGCCAA AATTATCAATCAGCTTAGCCGGGCGGGCT TTGTCACCGC
nsrR V43A-Rev	GCGGTGACAAAGCCCGCCCGGCTAAGCTGATTGATAATTTT GGCCATATGATTACGGGACACGCCGTAGACTTCT GTCAC
nsrR V43M-Fw	GTGACAGAAGTCTACGGCGTGTCCCGTAATCATATGATGAA AATTATCAATCAGCTTAGC CGGGCGGGCTTTGTCACCGC
nsrR V43M-Rev	GCGGTGACAAAGCCCGCCCGGCTAAGCTGATTGATAATTTT CATCATATGATTACGGGACACGCCGTAGACTTCTGTC AC
nsrR V43E-Fw	GTGACAGAAGTCTACGGCGTGTCCCGTAATCATATGGAGAA AATTATCAATCAGCTTAGCCGGGCGGGCTTTGTCACCGC
nsrR V43E-Rev	GCGGTGACAAAGCCCGCCCGGCTAAGCTGATTGATAATTTT CTCCATATGATTACGGGACACGCCGTAGACTTCTGTCAC
nsrR V43I-Fw	GTGACAGAAGTCTACGGCGTGTCCCGTAATCATATGATCAA AATTATCAA TCAGCTTAGC CGGGCGGGCT TTGTCACCGC

nsrR V43I- Rev	GCGGTGACAAAGCCCGCCCGGCTAAGCTGATTGATAATTTT GATCATATGATTACGGGACACGCCGTAGACTTCTGTCAC

* Nucleotides for the site-directed mutation are highlighted.

Control **GSNO**

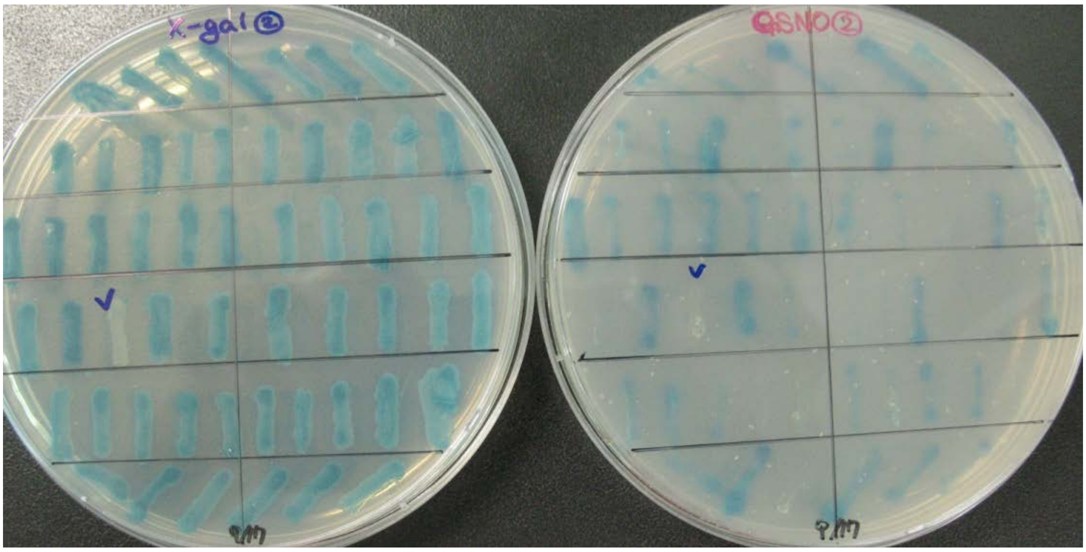

Fig. S1. Screening for the NsrR mutant clones that alter the *hmp-lacZ* expression. Colonies of *S.* typhimurium strains transformed with randomly mutated *nsrR* clones were replicated onto E minimal (0.2 % glucose) X-gal (25 mg ml^{-1}) plates in the absence (control) and presence of GSNO (250 μM) and incubated for 24 h. “V” denotes the colony harboring a mutant clone named as B4. A representative image is shown.

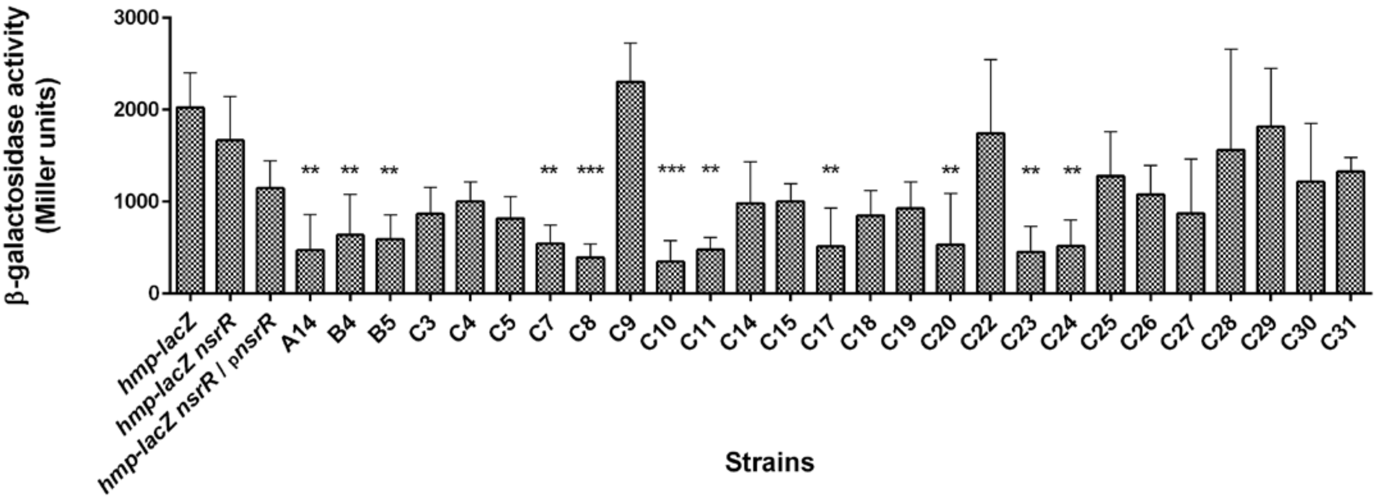

Fig. S2. The *hmp-lacZ* expression of the *nsrR* mutant clones selected through random mutagenesis. Overnight LB cultures of the selected *nsrR* mutant clones exhibiting repressive phenotypes in *hmp-lacZ* expression and bacterial growth on plates containing X-gal and GSNO were diluted 1:200 in EG media containing GSNO (250 μM) and cultured for 17 h. β-galactosidase activities were measured as described in Materials and Methods. Data shown are mean activity ± S.D. from three independent experiments. *P* value were determined by one-way analysis of variance. ***, $P < 0.001$; **, $P < 0.01$. All numbered clone strains share the same genotype, which includes *hmp-lacZ nsrR,* except for the mutant clone plasmid they harbor.

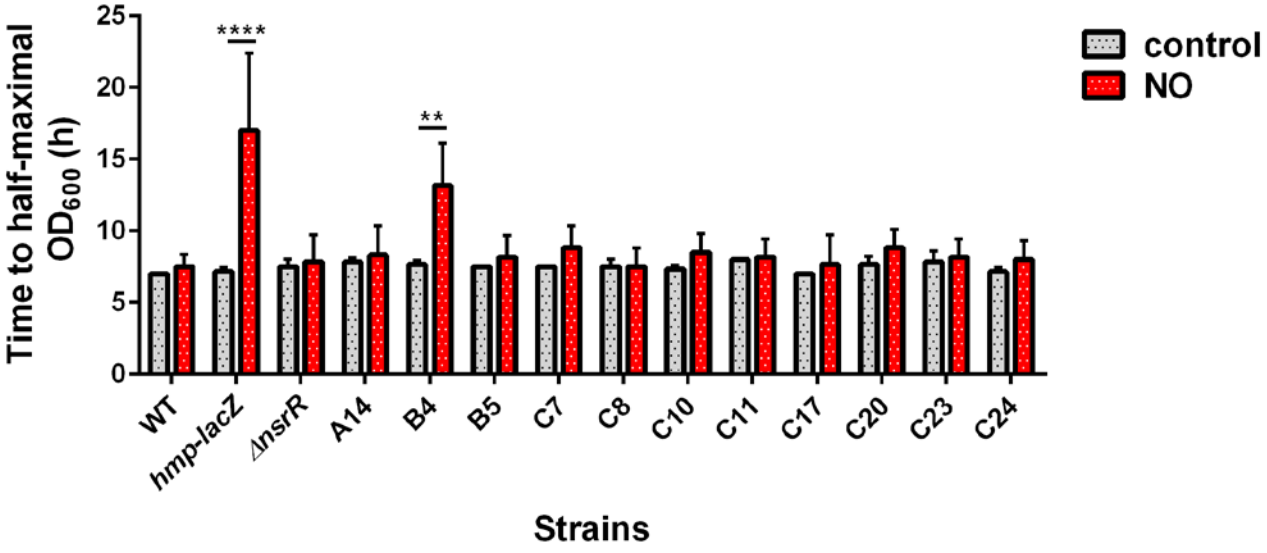

Fig. S3. The effect of *nsrR* mutant clones on the *S.* Typhimurium growth. The selected *nsrR* mutant clones, as identified by their reduced β-galactosidase activity in Fig. S2, were subcultured in EG media in the absence (control) and presence of GSNO (500 μM, NO) for 24 h, following the procedure described in the legend to Fig. S2. Bacterial growth is shown as the mean time ± S.D. to the half-maximal OD_{600} based on the growth curve for each strain, obtained from three independent experiments. *P* value were determined by two-way analysis of variance. ****, $P < 0.0001$; **, $P < 0.01$. All numbered clone strains share the same *ΔnsrR* genotype except for the mutant clone plasmid.

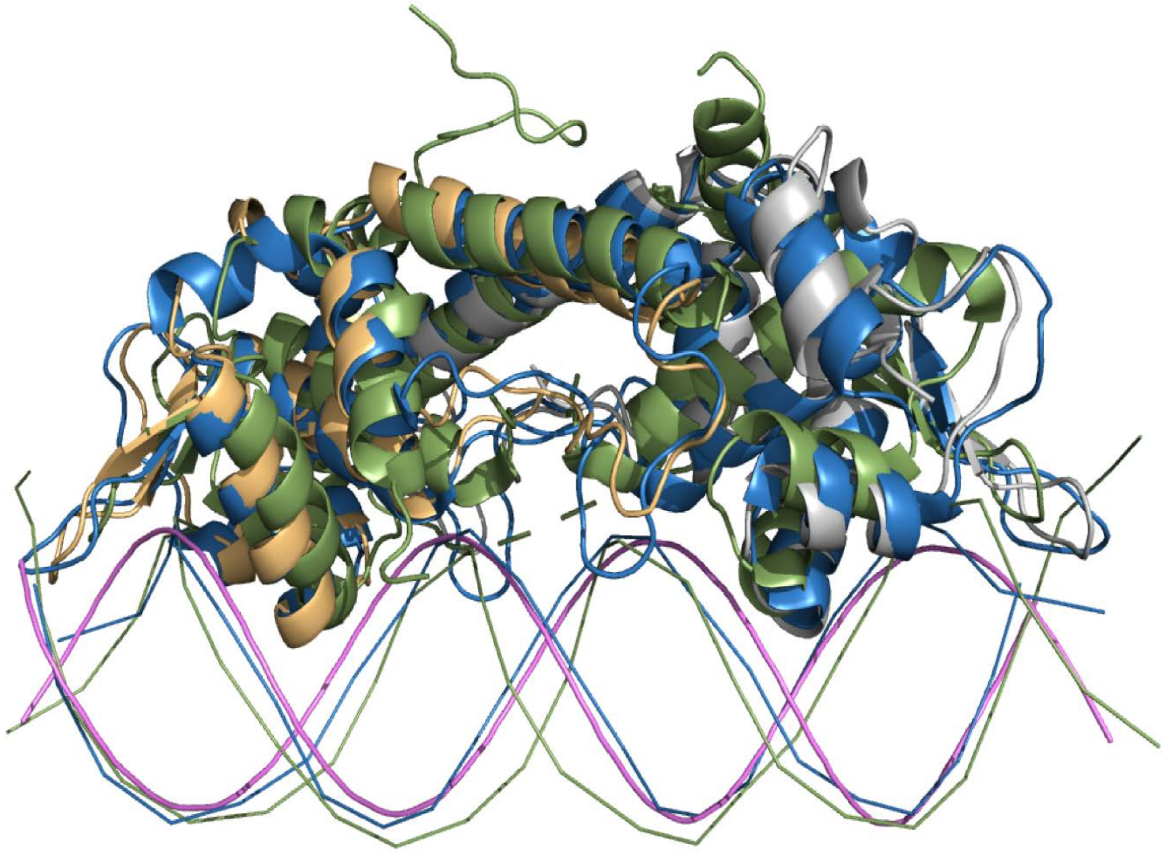

Fig. S4. Superposition of the predicted StNsrR-*hmp* complex structure with ScNsrR-*hmpA1* and EcIscR-*hya* complex structures. The predicted StNsrR monomers (gold and grey) and target *hmp* DNA helix (violet) structures were superposed with previously defined structures of ScNsrR-*hmpA1* (blue) and EcIscR-*hya* (green) complexes using the PyMol program. Ribbon models of proteins are shown, with their respective target DNA helices represented by lines.

Streptomyces coelicolor

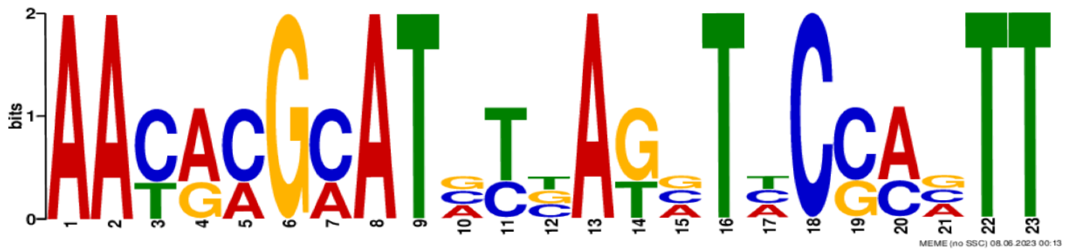


```
hmpA1   AACACGAATATCATCTACCAATT
hmpA2   AACAAGCATCTGAGATCCCAGTT
nsrR    AATGCGCATGCTAGGTTCGCCTT
```

***Salmonella* Typhimurium**

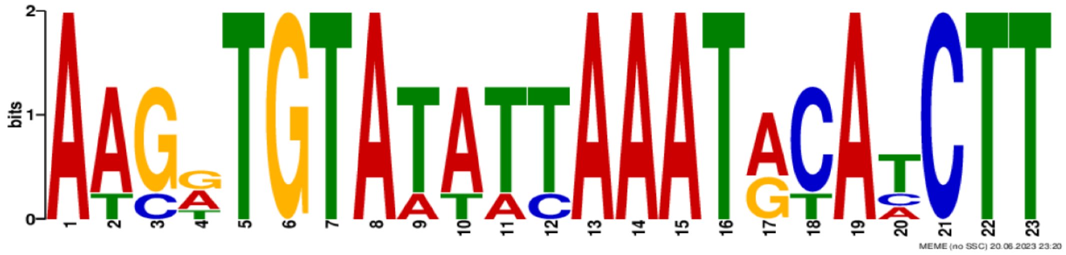


```
hcp-hcr   AACATGTATATTAAATATAACTT
STM1808   AAGGTGTATATTAAATACATCTT
hmp       AAGATGCATTTGATATACATCAT
ygbA      AAGGTGTAAAATAAATACACCTT
ytfE      AAGATGCATTTAAAATACAACTT
```

Fig. S5. NsrR binding consensus DNA sequences of *S. coelicolor* and *S.* Typhmurium. Logos of ScNsrR and StNsrR binding sites were determined by MEME analysis. The input DNA sequences used for analysis are listed below the logos. For ScNsrR binding sequences, DNA sequences of *hmpA1*, *hmpA2*, and *nsrR* genes identified by DNAse I footprint analysis were utilized. For StNsrR, highly putative NsrR binding sites of *hcp*, STM1808, *hmp*, *ygbA*, and *ytfE* genes in *S.* Typhimurium were selected based on previous studies, including transcriptome and *in silico* prediction analyses in *S.* Typhimurium and ChIP-Chip analysis in *E. coli* (8, 9).

References

1. Bang IS, Liu L, Vazquez-Torres A, Crouch ML, Stamler JS, Fang FC. 2006. Maintenance of nitric oxide and redox homeostasis by the salmonella flavohemoglobin hmp. J Biol Chem 281:28039-47.
2. McCollister BD, Bourret TJ, Gill R, Jones-Carson J, Vazquez-Torres A. 2005. Repression of SPI2 transcription by nitric oxide-producing, IFNgamma-activated macrophages promotes maturation of Salmonella phagosomes. J Exp Med 202:625-35.
3. Datsenko KA, Wanner BL. 2000. One-step inactivation of chromosomal genes in Escherichia coli K-12 using PCR products. Proceedings of the National Academy of Sciences of the United States of America 97:6640-5.
4. Poteete AR, Fenton AC. 1984. Lambda red-dependent growth and recombination of phage P22. Virology 134:161-7.
5. Cherepanov PP, Wackernagel W. 1995. Gene disruption in Escherichia coli: TcR and KmR cassettes with the option of Flp-catalyzed excision of the antibiotic-resistance determinant. Gene 158:9-14.
6. Guzman LM, Belin D, Carson MJ, Beckwith J. 1995. Tight regulation, modulation, and high-level expression by vectors containing the arabinose PBAD promoter. J Bacteriol 177:4121-30.
7. Blank, K., M. Hensel, and R.G. Gerlach, Rapid and highly efficient method for scarless mutagenesis within the Salmonella enterica chromosome. PLoS One, 2011. 6(1): p. e15763.
8. Karlinsey JE, Bang IS, Becker LA, Frawley ER, Porwollik S, Robbins HF, Thomas VC, Urbano R, McClelland M, Fang FC. 2012. The NsrR regulon in nitrosative stress resistance of Salmonella enterica serovar Typhimurium. Mol Microbiol 85:1179-93.

9. Partridge JD, Bodenmiller DM, Humphrys MS, Spiro S. 2009. NsrR targets in the Escherichia coli genome: new insights into DNA sequence requirements for binding and a role for NsrR in the regulation of motility. Mol Microbiol 73:680-94.
